# The impact on sleep of a multidisciplinary cognitive behavioural pain management programme: a pilot study

**DOI:** 10.1186/1471-2474-12-5

**Published:** 2011-01-10

**Authors:** Jennifer M Cunningham, Catherine Blake, Camillus K Power, Declan O'Keeffe, Valerie Kelly, Sheila Horan, Orla Spencer, Brona M Fullen

**Affiliations:** 1 UCD School of Public Health, Physiotherapy and Population Science, Health Science Centre, Belfield Campus, Dublin, Ireland; 2 Pain Service, Adelaide and Meath Hospital incorporating the National Children's Hospital, Tallaght, Dublin 24, Ireland; 3 Pain Service, St Vincent's University Hospital, Elm Park, Dublin 4, Ireland

## Abstract

**Background:**

Reduced sleep quality is a common complaint among patients with chronic pain, with 50-80% of patients reporting sleep disturbance. Improvements in pain and quality of life measures have been achieved using a multidisciplinary cognitive behavioural therapy pain management programme (CBT-PMP) that aims to recondition attitudes to pain, and improve patients' self-management of their condition. Despite its high prevalence in patients with chronic pain, there is very limited objective evidence for the effect of this intervention on sleep quality. The primary research objective is to investigate the short-term effect of a multidisciplinary CBT-PMP on subjective (measured by Pittsburg Sleep Quality Index) and objective sleep quality (measured by Actigraphy) in patients with chronic pain by comparison with a control group. The secondary objectives will investigate changes in function and mood, and then explore the relationship between objective and subjective sleep quality and physical and psychological outcome measures.

**Methods/Design:**

Patients who fulfil the inclusion criteria for attendance on the multidisciplinary CBT-PMP in the Adelaide and Meath Hospital, Tallaght, Dublin and are currently listed on the PMP waiting list will be invited to participate in this pilot study. Potential patients will be screened for sleep disturbance [determined by the Pittsburgh Sleep Quality Index (PSQI)]. Those patients with a sleep disturbance (PSQI >5) will be assigned to either the intervention group (immediate treatment), or control group (deferred treatment, i.e. the PMP they are listed for is more than six months away) based on where they appear on the waiting list. Baseline measures of sleep, function, and mood will be obtained using a combination of self-report questionnaires (the Hospital Anxiety and Depression Scale, the Short Form 36 health survey, the Pittsburgh Sleep Quality Index, the Tampa Scale for Kinesiophobia), and functional outcome measures. Sleep will be measured for seven days using actigraphy (Actiwatch 7). These measures will be repeated after the four week multidisciplinary cognitive behavioural therapy pain management programme, and at a two month follow-up. The waiting list control group will be assessed at baseline, and two months later. Analysis for the primary outcome will include between group differences of subjective and objective sleep parameters from baseline to follow-up using Independent T-tests or Mann-Whitney U tests. The secondary outcomes establishing relationships between the sleep variables and physical and psychological outcome measures will be established using multiple linear regression models.

**Discussion:**

This pilot study will evaluate the impact of a multidisciplinary CBT-PMP on both subjective and objective measures of sleep in patients with chronic pain and provide guidance for a larger clinical trial.

**Trial Registration:**

Current controlled trial ISRCTN: ISRCTN74913595

## Background

Chronic pain is a common disorder affecting 19% of the European population. It has a detrimental effect on all aspects of patient's quality of life: physically, psychologically, and socially [1]. Reduced sleep quality is a common complaint in this patient cohort with 50-80% reporting sleep disturbance [1,2]. Increasing evidence suggests a deteriorating cycle of pain and sleep. Pain can lead to poor sleep which in turn may result in increased next day pain, leading to further problems with next night sleep, as seen in other chronic pain conditions such as burns and fibromyalgia [3-6]. Whether cause or consequence, sleep disorders must be taken into account in the overall management of the patient in the same way as pain [7], as it has been hypothesized that better daytime pain control may lead to improved sleep quality [8]. Sleep disturbance has been shown to have a number of negative effects on both physical and psychological well-being. Physical effects include a reduction of physical functioning, lowered immune function, lower pain threshold [9], and an increase in the release of pro-inflammatory cytokines [10-13]. Psychologically, patient's mental capacity to manage pain is lowered, with increased risk of anxiety disorders and alcohol abuse [14-16]. Depression is also strongly associated with disturbed sleep patterns in this atient cohort [17-19].

International best practice for the management of chronic pain includes careful pharmacological management, promotion of activity, and the provision of a cognitive behavioural therapy pain management programme (CBT PMP) [20]. This multi-disciplinary intervention based on cognitive behavioural principles aimed at educating chronic pain patients on the physiology and psychology of chronic pain, healthy functioning, and the self-management of their pain problem. [21]. The programme includes daily clinical psychology (cognitive behavioural therapy, relaxation techniques), physiotherapy (progressive gym exercise programme), and occupational therapy (improving occupational function and environmental adaption).

The positive effect of this intervention on pain, disability, and mood has been established in two recent systematic reviews [22,23]. However, despite the known relationship between pain and sleep quality in this patient cohort, the impact of CBT-PMPs on sleep is limited. Significant positive changes in subjective sleep disturbances have been reported, however sleep was not the primary outcome measure, and no specific validated subjective or objective sleep quality measures were used [24]. A second study using both objective (actigraphy) and subjective outcome measures (PSQI) did report significant differences in sleep quality pre and post CBT PMP and at a three-month follow-up (P < 0.05) [25]. However, the main conclusions were drawn from the subjective data only. More recently a randomised controlled trial of individual cognitive behavioural therapy intervention for insomnia, delivered by a psychologist, demonstrated significant improvement in subjective and objective sleep indices for patients with chronic neck and back pain [26]. Drawing conclusions from subjective sleep quality outcome measures is limiting; objective and subjective sleep measures only correlate modestly with each other, therefore both should be included [27-30].

Due to the limited objective evidence to date, this pilot study will determine the short-term impact of a CBT-PMP on changes in objective and subjective sleep quality measures, as well as the impact of these changes on patient's psychological and physical measures.

## Method/Design

### Research Objectives

The primary research objective is to investigate the short-term multidisciplinary CBT-PMP on subjective (measured by PSQI) and objective sleep quality (measured by Actigraphy) in patients with chronic pain by comparison with a control group. The secondary objective will investigate changes in function and mood, and then explore the relationship between objective and subjective sleep quality, and physical and psychological outcome measures.

### Ethical Approval

This study was granted ethical approval from the Adelaide and Meath Hospital, Incorporating the National Children's Hospital Healthcare Group, Ethics and Medical Research Committee in October 2007.

### Study Design

**Figure 1:**
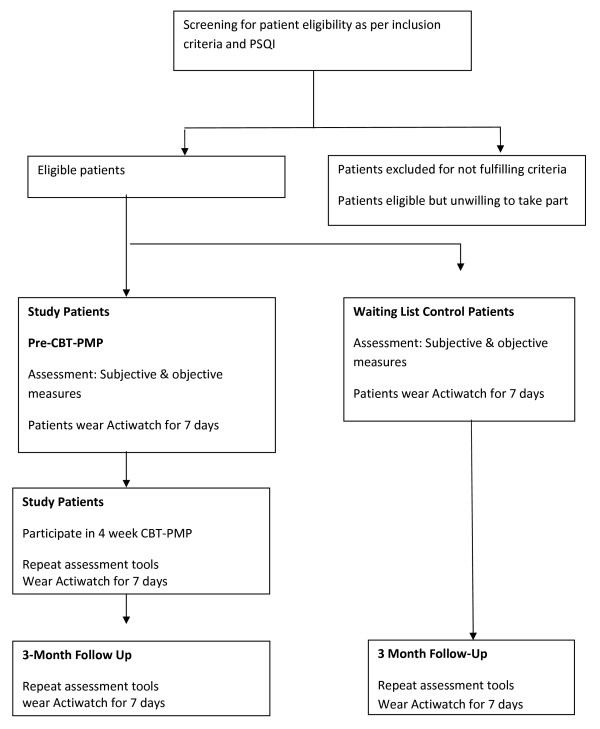
**Study Protocol**.

This study is a longitudinal pilot study, with patients recruited from the adult Pain service in the Adelaide and Meath Hospital, Dublin (Figure 1). Potential patients who fulfil the inclusion criteria for participation on the multidisciplinary CBT-PMP will be invited to participate in the study by the principle investigator (PI). The study protocol will be reviewed with potential participants, and all questions answered regarding the study. One week later, following a cooling off period, written consent will be obtained, and participants will complete the Pittsburgh Sleep Quality Index (PSQI). Those patients with a sleep disturbance (PSQI >5) will be assigned to either the intervention group (immediate treatment), or control group (deferred treatment, i.e. the PMP they are listed for is more than six months away) based on where they appear on the waiting list. Group membership will be concealed from the PI. To be eligible to take part in the study, participants must fulfil the following criteria:

#### Inclusion Criteria

• Deemed suitable for the AMNCH multidisciplinary CBT-PMP as per the multidisciplinary pain team

• Willing to participate in the study

• > 18 years old

• Suffering from sleep disturbance due to pain as determined by the PSQI questionnaire

• (a score >5 indicating sleep disturbance)

#### Exclusion Criteria

• Deemed unsuitable for the AMNCH multidisciplinary CBT-PMP as per the multidisciplinary pain team

• Unwilling to take part in the study

• <18 years old

• Do not suffer from sleep disturbance due to pain as determined by the PSQI questionnaire (a score >5 indicating sleep disturbance)

### Intervention

A multi-disciplinary CBT-PMP provides the patient with multiple therapies involving comprehensive rehabilitation in each of the specialized areas [31]. The core multi-disciplinary team includes a pain management physician, an occupational therapist, a physiotherapist and a clinical psychologist. They identify and change unhelpful thoughts and beliefs; promote relaxation, and help to change habits that contribute to disability [21,22].The multi-disciplinary team focuses on specific achievable goals established between the individual therapist and the patient [32]. Participants are advised to practice the skills they have learned both at home, and in other environments, integrating them into their everyday lives in order to help them to manage their pain more effectively [21].

Participants will attend three days a week for four consecutive weeks. The multidisciplinary programme includes daily two-hour group sessions with physiotherapy (stretching programme, core stability strengthening programme, paced individual exercise on a range of gym equipment, and functional restoration), occupational therapy (improving occupational function and environmental adaption), and clinical psychology (cognitive behavioural therapy, relaxation techniques). Weekly education sessions with the Pain consultant are also held.

### Assessment

A combination of valid and reliable self-report measures of pain, sleep quality, quality of life, nd mood, as well as objective measures of function and sleep will be used.

#### Study Patients

One week prior to the commencement of the multidisciplinary CBT-PMP patients will be given an appointment with the PI to obtain baseline measures. This will involve completing the self-report questionnaires and undergoing the physical tests. Patients will be instructed to wear the Actiwatch for seven days (24 hours a day), and to complete the Pittsburgh Sleep diary (PghSD) during this time. Following the four week CBT-PMP the patients will be reassessed, given the Actiwatch to wear for seven days, and complete the sleep diary. Two months later, one week prior to the appointment date patients will be sent (by registered post) the Actiwatch to wear for seven days, and the PghSD to complete. During the appointment patients will repeat the battery of self-report and functional tests.

#### Control Group

Patients will undergo the same assessment procedures at baseline and three months later.

### Instrumentation and Measurements

The instrumentation and measurement tools will include:

#### Self reported outcome measures

##### Numerical Rating Scale (NRS)

This 11-point scale (0-10 where 0 is no pain at all, and 10 is the worst pain imaginable) is ommonly used in the assessment of pain [33], and is a core outcome measure in clinical rials of chronic pain patients [34,35].

##### Tampa Scale for Kinesiophobia (TSK-II)

This scale measures the degree of fear of movement and/or re-injury, and is commonly used in acute and chronic pain populations [36]. It consists of an 11-item self-report questionnaire scored on a four-point Likert scale. The TSK-II consists of two subscales; a somatic focus subscale (items 3, 4, 6, 7, and 10) that reflect the beliefs that there is something seriously wrong with the body, and an activity avoidance subscale (items 1, 2, 5, 8, 9, and 11) that reflects the beliefs that avoiding exercise or physical activities might prevent an increase of pain. A reduction of at least four points on both measures maximises the likelihood of correctly identifying an important reduction in fear of movement. The reliability of the TSK-11 has been established [36].

##### SF-36 Health Survey

This tool will be used to measure health related quality of life (HRQL) and physical functioning [37]. It consists of 36 items that measures 8 multi-item variables including physical functioning, social functioning, role limitations due to physical problem, mental health, energy and vitality, pain and general perception of health, there is also one extra un-scaled item to assess a patient's perceived change in health in the last year. The SF36 is a validated and reliable tool with a high internal reliability value across all items >0.7 except those in the social dimension which were found to be >0.5 [38,39]

##### The Hospital Anxiety and Depression Scale (HADS)

This questionnaire will be used to assess a patient's level of anxiety and depression. It is divided into two subscales; an anxiety subscale (HAD-A) and a depression subscale (HADS-D). Both subscales contain 7 intermingled items. Scores of 11 or more on either subscale are considered to represent a significant case of psychological morbidity, scores of 8-10 represents borderline psychological morbidity and scores of 0-7 represent normal levels of anxiety and/or depression [40]. The HADS has high mean levels of internal consistency HAD-A 0.83 and HAD-D 0.82 (Cronbach's alpha), and a sensitivity and specificity of 0.8 for both HAD (A) and HAD (D)[41].

##### Pittsburgh Sleep Diary (PghSD)

This tool will be used as an adjunct for the Actiwatch AW7 to give a written recording for the objective Actiwatch data. It will be filled in every day for the 7 days that the patient wears the Actiwatch. The instrument comprises two separate sections; a bedtime component that will be filled in just before bed, and a wake time component that will be filled in first thing in the morning. The bedtime component documents events preceding that nights' sleep that may have an impact on sleep quality or quantity; the number of caffeinated drinks consumed, or the number of daytime naps. The wake time component documents events that occur during the night that may impact on that night's sleep such as the number of wake bouts during the night, and patient satisfaction of sleep quality. The PghSD measures of both sleep timing and sleep quality has been shown to have correlations between 0.56 and 0.81 (n = 39, P < 0.001) [42].

### Sleep

#### Sleep

Objective sleep quality will be measured using the Actiwatch AW7 (Cambridge Neurotechnology). Whist not as accurate as the gold standard of sleep assessment, actigraphy has been shown to correlate reasonably well with polysomnography; with intraclass correlation co-efficient of 0.76 for total sleep time, 0.61 for sleep efficiency, and 0.58 for both wake after sleep onset and sleep fragmentation index measurements [43]. This compact and lightweight electronic device similar in size to a wrist-watch is worn on the non-dominant hand, and measures and records physical movement. It automatically collects and scores data for seven sleep quality parameters: wake after sleep onset percentage, sleep onset latency, actual sleep percentage, mean night-time activity, fragmentation index, number of wake bouts, and sleep efficiency. This movement data is then down loaded and analysed using Actiwatch 7 software. Activity is measured in counts; the number of counts is proportional to the intensity of the movement. The peak intensity of the movement in each second is summed into a user selectable epoch (range two seconds and 15 minutes). Data collected is more accurate with a shorter epoch length [44]. The current study proposes to record data for 7 days using a 30 second epoch length, which has been shown to be adequate for the assessment of sleep disturbance [44].

When a patient is ready to sleep they will press the button on the actiwatch to signal this intention of sleep start. When the patient wakes they will press the button a second time to indicate that sleep has finished, this defines the sleep period for analysis. If a patient forgets to press the button, the Pittsburgh sleep diary will be used to determine the sleep start and/or finish. The actiwatch 7 sleep software can then determine the objective sleep quality outcome measures: wake after sleep onset percentage, sleep onset latency, actual sleep percentage, mean night-time activity, fragmentation index, number of wake bouts, and sleep efficiency.

#### Function

The Simmond's functional assessment tool [45] is a validated battery of physical performance measures designed to assess function in patients with chronic LBP. For the purpose of the current study two of these physical performance measures will be included. The sit-to-stand test (STS) times patients as they get up and down out of a chair five times, repeated twice, and the mean time is recorded. The 360° rollover tests times the patient rolling 360 degrees. The Simmond's battery of tests has been found to have good validity and reliability of between 0.69 and 0.99, with a construct validity of P < 0.0001 [46].

### Data management

All participant data will be coded to ensure anonymity, and stored in a password protected computer, and questionnaires locked in a filing cabinet at the School of Public Health, Physiotherapy and Population Science, University College Dublin, Ireland.

### Sample size/Power calculation

While formal power based calculations are not be necessary for a pilot trial, sample estimates to guide recruitment to this study were based on existing data, using objective sleep disturbance as the primary outcome measure. Actigraphy data from patients with chronic LBP [47] indicated that sleep efficiency percentage (mean = 77.8, Sd 7.8) was the variable which differed most significantly from controls. On this basis, a minimum of 90 subjects will be required to detect a moderate treatment effect (ES 0.6). For the purposes of establishing feasibility, and providing data to inform the design of a future main trial based on our CBT-PMP population, we however aim to recruit 24 patients for each arm of the pilot study.

### Data Analysis

All data will be cleaned and entered into the Statistical Package for the Social Sciences (SPSS, Version 16) for analysis. Baseline and demographic data will be presented using descriptive statistics. Differences from baseline will be calculated for all primary and secondary outcomes. Mean differences, standard deviations and 95% confidence intervals will be calculated for continuous variables. Data will be tested for assumptions of normality and parametric analysis will be performed where this is shown. Non-parametric tests will be performed on all ordinal and non-normally distributed data. All tests will be 2 sided with a critical value of p < 0.05.

Primary Outcome: Between group differences in changes to the primary outcome (objective and self-report sleep measures) from baseline to follow up will be performed with independent t tests or Mann Whitney U tests. An intention to treat analysis will also be performed, where missing data are estimated using regression based multiple imputation methods.

Secondary Outcomes: To establish relationships between the sleep variables and other outcome measures (physical and psychological), multiple linear regression models will be constructed. Univariate regression will first be performed and those variables shown to be associated with the dependent variable will be included in the multivariate model. Backward regression will be used to determine the model of best fit.

## Discussion

This pilot study will determine the impact of a multidisciplinary CBT-PMP on objective and self-report sleep quality in patients with chronic pain. It will also investigate the relationship between sleep variables and physical and psychological outcome measures. These results will add to the knowledge of the impact of a multidisciplinary CBT-PMP on patient's quality of life.

## Abbreviations

AMNCH: Adelaide and Meath Hospital Dublin Incorporating the National Children's Hospital; CBT PMP: Cognitive Behavioural Therapy Pain Management Programme, HADS: Hospital Anxiety and Depression Scale; LBP: Low Back Pain, NRS: Numerical Rating Scale; PghSD: Pittsburgh Sleep Diary; PI: Principal investigator; PSQI: Pittsburgh Sleep Quality Index; QOL: Quality of Life; STS: Sit to stand; SPSS: Statistical Package for Social Sciences; TSK: Tampa Scale of Kinesiophobia

## Competing interests

This study was funded by an unrestricted educational grant from Pfizer. The authors have no competing interests.

## Authors' contributions

JC is the principal investigator. JC and her supervisory team of BMF, CB, CKP, DOK designed the study and were responsible for the protocol. CB is responsible for the sample size calculation for the design of the statistical analysis, and the evaluation of the database. VK, SH, and OS delivered the cognitive behavioural pain management programme. All authors read and approved the final manuscript.

## Pre-publication history

The pre-publication history for this paper can be accessed here:

http://www.biomedcentral.com/1471-2474/12/5/prepub
